# Insufficient Sleep Duration and Overweight/Obesity among Adolescents in a Chinese Population

**DOI:** 10.3390/ijerph15050997

**Published:** 2018-05-15

**Authors:** Qing-Hai Gong, Si-Xuan Li, Hui Li, Jun Cui, Guo-Zhang Xu

**Affiliations:** Ningbo Center for Disease Control and Prevention, Ningbo 315010, China; gongqinghai@163.com (Q.-H.G.); lisx@nbcdc.org.cn (S.-X.L.); lih@nbcdc.org.cn (H.L.); cuij@nbcdc.org.cn (J.C.)

**Keywords:** sleep duration, BMI, overweight/obesity, adolescents, China

## Abstract

Objectives: The objective of this study was to investigate the relationship between sleep duration and overweight/obesity among Chinese adolescents. Methods: A school-based cross-sectional study was conducted among Chinese adolescents in 2016. In total, 2795 school-aged Chinese children aged 12 to 13 years participated in this study. Participants were asked to complete self-administered surveys during a 45-min class period in their classroom. Details of the questionnaire about health-related behaviors included sleep habits, physical activity, screen time, cigarette use, and alcohol use. Height, weight, waist circumference and hip circumference were directly measured. Results: The mean sleep duration was 8.7 h/day. In total, 43.0% of the participants had a sleep duration of less than 9 h/day. Sleep duration was significantly inversely related to BMI, waist circumference, hip circumference and WHtR in multiple linear regression analyses in both genders. Logistic regression models showed that insufficient sleep (<9 h/day) was associated with high odds of overweight/obesity among both young boys and girls. Conclusions: Insufficient sleep duration was associated with overweight/obesity in Chinese adolescents, and short sleep duration was probably associated with central adiposity, especially among boys.

## 1. Introduction

Obesity or overweight is a major public health issue in China. According to the China Health and Nutrition Survey, 22.1% of Chinese adolescents aged 7–17 years were either overweight or had obesity [1,2]. Overweight or obesity has been shown to be associated with an increased risk of diabetes, cardiovascular disease, cancer and premature death [3,4]. Sleep, along with physical activity, diet, genetic factors, environmental pollution, parents’ education and socio-economic status, plays an important role in health [5,6,7,8,9,10,11]. A number of studies have reported that insufficient sleep or short sleep duration was related to an increased risk of overweight and obesity in both adults and children [12,13]. Moreover, the sleep-obesity association may vary by race and age [14,15]. However, some studies have also reported conflicting associations between sleep duration and Body Mass Index (BMI) and/or anthropometric measures [16,17,18]. Thus, the relationship between sleep duration and obesity or/and anthropometric measures remains uncertain.

In recent years, sleep curtailment has become a hallmark of modern society. Over the past century, sleep duration has decreased by 0.75 min nightly per year in children and adolescents [19]. It has been reported that roughly 40% of school-aged children are not getting enough sleep [20]. A recent cross-sectional study in Sweden indicated that approximately 40% of children (aged 10 years) had sleep duration of less than 9 h per day [21]. Similarly, our previous epidemiological study also found that roughly 40% of sampled adolescents had a sleep duration of less than 8 h per day in Ningbo, Zhejiang province, China [22]. Targeting insufficient sleep duration may offer a novel and effective method for the prevention and treatment of obesity [23].

The objective of this study was to investigate the relationship between sleep duration and overweight/obesity among Chinese adolescents by means of a school-based cross-sectional study. After controlling for demographic, socioeconomic and physical activities, we hypothesized that sleep duration would be negatively associated with BMI, waist circumference, hip circumference and waist-to-height ratio (WHtR).

## 2. Methods

### 2.1. Study Subjects

This study used data from the 2016–2018 Ningbo Youth Risk Behavior Survey (NYRBS), a school-based prospective study in Ningbo, Zhejiang province, China. The present study included a questionnaire survey and a physical examination. From October to November 2016, 13 middle schools from 10 districts of Ningbo were recruited by using purposeful convenience sampling methods to participate in this study. In total, 2795 first-grade-in-junior-high schoolchildren aged 12 to 13 years participated in this study.

### 2.2. Ethical Aspects

This study was approved by the ethics committee of Ningbo Center for Disease Control and Prevention (NO. 201606). Prior to the study, parents (or legal guardians) of all participants were asked to sign a written informed consent form for their children. 

### 2.3. Measures of Sleep and Health-Related Behaviors

In our study, all participants were asked to complete self-administered surveys during a 45-min class period in their classroom. The questionnaire was established with reference to the Youth Risk Behavior Surveillance (YRBS) survey conducted in the United States [24]. Sleep duration was assessed by the following questions, “On an average morning, what time do you get up?” “On an average evening, what time do you go to bed?” All participants reported their time of going to bed and the time of arising. Sleep duration was calculated according to the following equation: sleep duration = (get up time + 24) − (go to bed time) [25]. According to the U. S. National Sleep Foundation and other epidemiology studies, sleep durations were dichotomized as insufficient sleep (<9 h/day) and sufficient sleep (≥9 h/day) for children ages 7–13 years [25,26,27]. Details of the questionnaire relating to other health-related behaviors, including physical activity, screen time, cigarette use, and alcohol use, were reported in our previous study [22].

### 2.4. Anthropometric Measurements

Participants fasted the night before and were measured the next morning by trained research staff. All staff used the same devices, which were calibrated at the time of measurement. The height and weight of adolescents were measured with thin clothes (shorts and T-shirts) and without shoes. A waist circumference measurement was taken at the level of the umbilicus to the nearest 0.1 cm. Measurement for hip circumference was taken at maximum circumference of the buttocks to the nearest 0.1 cm. Height was measured to the nearest 0.1 cm with a free-standing stadiometer mounted on a rigid tripod (GMCS-I, Xindong Huateng Sports Equipment Co. Ltd., Beijing, China). Fasting body weight was measured to the nearest 0.1 kg on a digital scale (RGT-140, Weighing Apparatus Co. Ltd., Changzhou Wujin, China). BMI was calculated as weight in kilograms divided by height in meters squared (kg/m^2^). All anthropometric values consisted of the mean of three measures. Obesity and overweight were defined according to the International Obesity Task Force (IOTF) definition [28]. Central adiposity was defined as WHtR ≥ 0.50 [29]. All the questionnaire surveys and measurements were conducted at 10:00–11:30 a.m. on week days.

### 2.5. Statistical Analysis

Continuous variables were summarized as means ± standard deviation (SD), and categorical variables were summarized as percentages. Differences in continuous variables were examined using the *t*-test, whereas categorical variables were analyzed using a chi-square test. A multiple linear regression analysis was used to evaluate the association between the sleep duration and BMI, waist circumference, hip circumference and WHtR. Logistic regression analysis was performed to estimate the odds ratios (ORs) and 95% confidence intervals (CIs) of overweight and obesity by sleep duration adjusted for potential confounders. All the tests were two-sided, and the significance level was set at 0.05. All analyses were performed with the Statistical Package for Social Sciences (SPSS) for Windows, version 17.0 (SPSS Inc., Chicago, IL, USA).

## 3. Results

A total of 2901 students participated in this study, 106 subjects were excluded because of missing values. Ultimately, 2795 participants were included in the analysis. Among 2795 adolescents aged 12–13 years (mean 12.6 ± 0.55, 51.5% boys), the prevalence of overweight and obesity was 16.6% and 3.6%, respectively. The mean sleep duration was 8.7 ± 0.85. In total, 43.0% of the participants had sleep duration of less than 9 h/day. 

When sleep duration was dichotomized as either insufficiency (<9 h/day) or sufficiency (≥9 h/day), insufficient sleepers showed significantly higher BMI (*p* < 0.001), hip circumference (*p* < 0.001), waist circumference (*p* < 0.001) and WHtR (*p* = 0.024). Prevalence of overweight, obesity, felt sad or hopeless, current drinking, and breakfast skipping were significantly higher in insufficient sleepers than in sufficient sleepers (all *p* < 0.05) (Table 1). 

Table 2 shows the multiple linear regression analysis of sleep duration and BMI, waist circumference, hip circumference and WHtR for each gender. In total, sleep duration was significantly associated with BMI (β = −0.411, *p* < 0.001), waist circumference (β = −0.900, *p* < 0.001), hip circumference (β = −1.063, *p* < 0.001) and WHtR (β = −0.004, *p* < 0.001) after being adjusted for age, gender, parents’ marriage status, parents’ highest education at college level, current smoking, current drinking, breakfast skipping, perceived sad/hopeless, watching television, and physical activity. For boys and girls, sleep duration showed significant inverse relationships with BMI (β = −0.344, *p* < 0.001 and β = −0.499, *p* < 0.001, respectively), waist circumference (β = −1.098, *p* < 0.001 and β = −0.695, *p =* 0.006, respectively), hip circumference (β = −0.975, *p* < 0.001 and β = −1.193, *p* < 0.001, respectively), and WHtR (β = −0.004, *p* < 0.001 and β = −0.004, *p =* 0.022, respectively) after being adjusted for age, parents’ marriage status, parents’ highest education at college level, current smoking, current drinking, breakfast skipping, perceived sad/hopeless, watching television and physical activity.

The relationship between the insufficient sleepers (sleep duration <9 h/day) and overweight/obesity was examined with multivariate logistic regression analyses by gender (Table 3). After adjustment for age, gender, parents’ marriage status, parents’ highest education at college level, current smoking, current drinking, breakfast skipping, perceived sad/hopeless, watching television and physical activity, insufficient sleep was significantly associated with increased odds of obesity/overweight (AOR = 1.474, 95% CI = 1.212 to 1.792, *p* < 0.001) (Table 3). For both boys and girls, logistic regression models showed that insufficient sleep was associated with increased odds of adolescents’ overweight/obesity in the model adjusted for age, parents’ highest education at college level, current drinking, breakfast skipping, perceived sad or hopeless (AOR = 1.513, 95% CI = 1.180 to 1.940, *p =* 0.001 and AOR = 1.405, 95% CI = 1.021 to 1.934, *p =* 0.038, respectively). Table 3 also shows that sleep duration (a continuous variable) was negatively associated with overweight/obesity in the adjusted model for both boys and girls (AOR = 0.806, 95% CI = 0.701 to 0.928, *p =* 0.003 and AOR = 0.739, 95% CI = 0.615 to 0.887, *p =* 0.001, respectively), suggesting that with a 1-h increase in sleep duration among both boys and girls, obesity/overweight risks decreased by more than 20%. Table 4 presents the results of multivariate logistic regression analyses regarding associations between sleep duration and central adiposity (WHtR > 0.50). Logistic regression models showed that sleep duration (a continuous variable) was negatively associated with central adiposity (WHtR ≥ 0.50) in the adjusted model for boys but not for girls (AOR = 0.788, 95% CI = 0.667 to 0.931, *p =* 0.005 and AOR = 0.858, 95% CI = 0.669 to 1.101, *p =* 0.228, respectively).

## 4. Discussion

In this large, school-based study, insufficient sleep duration was associated with overweight/obesity among adolescents aged 12–13 years living in Ningbo. Adjusting for demographic characteristics, parents’ marriage status, parents’ educational status and lifestyle factors did not change this association, while the associations were consistent among boys and girls. Compared with sufficient sleepers (≥9 h/day), insufficient sleepers (<9 h/day) had a 47% higher risk of being overweight/obese. The results of our study also indicated that with a 1-h increase in sleep duration among adolescents, the obesity/overweight risk decreased by more than 20%. Most population-based studies linking the relationship between sleep duration and obesity have used body mass index (BMI) based on self-reported height and weight, which could result in some biases [30]. This study is one of the first to investigate the sleep-obesity relationships in Chinese adolescents using direct anthropometric measurements. A previous study reported that, compared with long sleep (≥11 h/night), short sleep (<9 h/night) was associated with increased odds ratio for obesity among Chinese preschool children aged 3–4 years [31]. Similarly, a pervious study conducted among Chinese children aged 7–12 years found that, compared with sleep duration ≥10 h/day, sleep duration <9 h/day increased the likelihood of overweight/obesity [32]. Taken together, these findings support the notion that insufficient sleep is a risk factor for overweight and obesity in preschool and adolescent children. 

Interestingly, our study found that sleep duration was significantly associated with adolescents’ overweight/obesity even after adjusting for age, parents’ marriage status, parents’ highest education at college level, current smoking, current drinking, breakfast skipping, perceived sad/hopeless, watching television and physical activity. It has been proposed that gender, depression or sadness, parents’ educational status, physical activity and drinking could confound the associations between sleep duration and overweight/obesity [20,33,34,35]. However, this view was not supported by our findings. Our study may further underscore that sleep duration is an independent risk factor for overweight and obesity. 

Little is known about the relationship between sleep duration and anthropometric measures among Chinese adolescents. A recent study found that short sleep duration was positively associated with BMI in both young men and women, and was positively associated with waist circumference among young women, but not among men [36]. Another cross-sectional study reported that short sleep duration was significantly associated with high adiposity measures among Chinese adolescents [37]. It has been recommended that waist circumference, hip circumference or WHtR should be used as measures of central adiposity [38,39]. However, there are no commonly accepted cut-offs for children’s central adiposity based on waist circumference, hip circumference or WHtR. Our results showed that total sleep duration was associated with BMI, waist circumference, hip circumference and WHtR in an inverse linear fashion in both boys and girls. In addition, waist circumference and hip circumference were more strongly associated than BMI with sleep duration in both genders. Our study indicated that short sleep duration may be associated with central adiposity, especially among boys. To the best of our knowledge, there are no data showing an association between waist circumference, hip circumference, WHtR and sleep duration in Chinese children or adolescents. 

Potential mechanisms of insufficient sleep and BMI or obesity are not fully understood. Several mechanisms could explain part of this association, including increased levels of ghrelin, decreased levels of leptin, and increased hunger and appetite [40,41,42]. In epidemiological studies, inadequate sleep duration was related to lower physical activity and lower fruit and vegetable consumption [22,43]. Summarized together, insufficient sleep may affect energy homeostasis, including energy expenditure and the regulation of hunger and appetite, thereby leading to increased weight gain and overweight/obesity. 

There are some limitations of this study. First, due to the cross-sectional nature of the study design, the temporal relationship between sleep duration and obesity cannot be determined. Second, self-reported sleep duration may introduce measurement error [44]. However, this measurement error was more likely to be random, and there was no evidence that overweight or obesity tended to report shorter sleep. Furthermore, we did not measure sleep duration on weekdays and weekends separately. Therefore, we cannot know whether sleep duration plays different roles between weekdays and weekend days. Finally, this study did not measure dietary habits and other aspects of sleep, such as sleep quality, social jetlag, which may also be shown to be related to the risk of obesity. Strengths of our study include the large sample size and the fact that BMI, waist circumference and hip circumference were objectively measures. 

## 5. Conclusions

This study demonstrates that insufficient sleep duration could be associated with overweight/obesity, and a per-hour increase in sleep duration could be associated with overweight/obesity in Chinese adolescents. Furthermore, short sleep duration may be associated with central adiposity, especially among boys. Sleep has been recognized as an important amendable risk factor for obesity. Increasing sleep duration among adolescents may play an important role in prevention and treatment of obesity. Further research, longitudinal and intervention studies are needed to confirm causal pathways underlying the association between sleep duration and overweight/obesity among adolescents. 

## Figures and Tables

**Table 1:** Characteristics of the study subjects by sleep duration category (*n* = 2795).

Variables	Sleep Duration		*p*
	Sufficient Sleep ≥ 9 h (*n* = 1593)	Insufficient Sleep < 9 h (*n* = 1202)	
Age, years, mean (SD ^a^)	12.38 ± 0.57	12.34 ± 0.53	0.113
Gender (%)			<0.001
Male	55.9	48.3	
Female	44.1	51.7	
Parents’ highest education at college level (%)			<0.001
Both had college degree	9.4	14.2	
Only one of them had college degree	10.4	13.4	
None of them had college	80.2	72.4	
Parents’ marriage status (%)			0.440
Married	90.1	91.0	
Divorced/widowed/separated	9.9	9.0	
Bedtimes, mean (SD ^a^) (hh:ss)	20:49(0:32)	21:43(0:37)	<0.001
Get up time, mean (SD ^a^) (hh:ss)	6:16(0:35)	5:54(0:23)	0.013
BMI ^b^, mean (SD ^a^) (kg/m^2^)	18.84 ± 3.27	19.38 ± 3.52	<0.001
WHtR ^c^, mean (SD ^a^)	0.42 ± 0.05	0.43 ± 0.05	0.024
Hip Circumference, mean (SD ^a^) (cm)	84.51 ± 7.75	86.35 ± 8.08	<0.001
Waist Circumference, mean (SD ^a^) (cm)	67.57 ± 8.93	68.77 ± 9.18	<0.001
Weight status (%)			0.001
Under/Normal weight	82.9	77.3	
Overweight	14.1	18.6	
Obese	3.0	4.1	
Perceived sad/hopeless ^†^			<0.001
NO	86.9	81.9	
YES	13.1	18.1	
Current smoking ^‡^			0.536
No	97.9	98.2	
Yes	2.1	1.8	
Current drinking ^‡^			0.002
No	89.2	85.2	
Yes	10.8	14.8	
Breakfast skipping ^†^			<0.001
No	79.0	70.6	
Yes	21.0	29.4	
Physical activity ^‡^			
Moderate physical activity (≥1 d/week)			0.282
No	7.3	8.4	
Yes	92.7	91.6	
Muscle strengthening activity (≥1 d/week)			0.196
No	38.9	41.4	
Yes	61.1	59.6	
Watching television ≥ 2 h ^†^			0.371
No	75.5	76.9	
Yes	24.5	23.1	

^a^ SD, standard deviation; ^b^ BMI, body mass index; ^c^ WHtR, waist-to-height ration; ^†^ during the past 12 months; ^‡^ during the past 30 days.

**Table 2:** Multivariate linear regression analyses of sleep duration and obesity-related variables.

		Sleep Duration Time		
		β	SE ^a^	*p*
Both genders (*n* = 2795) Adjusted for model 1				
	BMI ^b^ (kg/m^2^)	−0.411	0.077	<0.001
	Waist Circumference (cm)	−0.900	0.202	<0.001
	Hip Circumference (cm)	−1.063	0.178	<0.001
	WHtR ^c^	−0.004	0.001	<0.001
Boys (*n* = 1440) Adjusted for model 2				
	BMI ^b^ (kg/m^2^)	−0.344	0.108	0.002
	Waist Circumference (cm)	−1.098	0.300	<0.001
	Hip Circumference (cm)	−0.975	0.251	<0.001
	WHtR ^c^	−0.005	0.002	0.009
Girls (*n* = 1355) Adjusted for model 2				
	BMI ^b^ (kg/m^2^)	−0.499	0.109	<0.001
	Waist Circumference (cm)	−0.695	0.264	0.006
	Hip Circumference (cm)	−1.193	0.254	<0.001
	WHtR ^c^	−0.003	0.002	0.022

^a^ SE, Standard Error; ^b^ BMI, body mass index; ^c^ WHtR, Waist-to-height ration; Model 1: adjusted for: age, gender, parents’ marriage status, parents’ highest education at college level, current smoking, current drinking, breakfast skipping, perceived sad/hopeless, watching television and physical activity; Model 2: adjusted for: age, parents’ marriage status, parents’ highest education at college level, current smoking, current drinking, breakfasts kipping, perceived sad/hopeless, watching television and physical activity.

**Table 3:** Association between sleep duration and overweight/obesity.

Sleep Duration	Crude		Model 1		Model 2	
	OR (95% CI)	*p*	OR (95% CI)	*p*	OR (95% CI)	*p*
Both genders (*n* = 2795)						
Insufficient sleep	1.425(1.178 to 1.742）	<0.001	1.483(1.223 to 1.799）	<0.001	1.474(1.212 to 1.792）	<0.001
Sufficient sleep	1		1		1	
Per hour	0.780(0.699 to 0.871)	<0.001	0.767 (0.687 to 0.857)	<0.001	0.777 (0.695 to 0.868)	<0.001
Boys (*n* = 1440)						
Insufficient sleep	1.425(1.178 to 1.742）	<0.001	1.497(1.172 to 1.912）	0.001	1.513(1.180 to 1.940）	0.001
Sufficient sleep	1		1		1	
Per hour	0.780(0.699 to 0.871)	<0.001	0.809 (0.704 to 0.930)	0.003	0.806 (0.701 to 0.928)	0.003
Girls (*n* = 1355)						
Insufficient sleep	1.425(1.178 to 1.742）	<0.001	1.464(1.070 to 2.003）	0.017	1.405(1.021 to 1.934）	0.038
Sufficient sleep	1		1		1	
Per hour	0.780(0.699 to 0.871)	<0.001	0.702 (0.586 to 0.841)	<0.001	0.739 (0.615 to 0.887)	0.001

CI, Confidence interval; OR, odds ratio. Model 1: adjusted for age (in both gender, also adjusted for gender). Model 2: adjusted for: age, parents’ marriage status, parents’ highest education at college level, current smoking, current drinking, breakfast skipping, perceived sad/hopeless, watching television and physical activity (in both gender, also adjusted for gender).

**Table 4:** Association between sleep duration and central adiposity (WHtR ≥ 0.50).

Sleep Duration	Crude		Model 1		Model 2	
	OR (95% CI)	*p*	OR (95% CI)	*p*	OR (95% CI)	*p*
Both genders (*n* = 2795)						
Insufficient sleep	1.227 (0.967 to 1.557)	0.093	1.303(1.024 to 1.658)	0.031	1.293 (1.011 to 1.653)	0.040
Sufficient sleep	1		1		1	
Per hour	0.816 (0.712 to 0.935)	0.003	0.796 (0.694 to 0.912)	0.001	0.814 (0.710 to 0.934)	0.003
Boys (*n* = 1440)						
Insufficient sleep	1.313 (0.981 to 1.757)	0.067	1.304 (0.974 to 1.745)	0.075	1.310 (0.972 to 1.766)	0.076
Sufficient sleep	1		1		1	
Per hour	0.780 (0.662 to 0.920)	0.003	0.782 (0.664 to 0.923)	0.004	0.788 (0.667 to 0.931)	0.005
Girls (*n* = 1355)						
Insufficient sleep	1.290 (0.841 to 1.979)	0.243	1.292 (0.842 to 1.983)	0.240	1.269 (0.819 to 1.967)	0.286
Sufficient sleep	1		1		1	
Per hour	0.827 (0.648 to 1.057)	0.129	0.826 (0.647 to 1.056)	0.127	0.858 (0.669 to 1.101)	0.228

CI, Confidence interval; OR, odds ratio. Model 1: adjusted for age (in both gender, also adjusted for gender). Model 2: adjusted for: age, parents’ marriage status, parents’ highest education at college level, current smoking, current drinking, breakfast skipping, perceived sad/hopeless, watching television and physical activity (in both gender, also adjusted for gender).
